# “I swear it’s not Tourette’s!” - it is time to rethink coprolalia in Tourette syndrome

**DOI:** 10.3389/fpsyt.2026.1948442

**Published:** 2026-10-02

**Authors:** Kirsten R. Müller-Vahl, Christelle Nilles, Nanette Mol Debes, Natalia Szejko, Tammy Hedderly

**Affiliations:** 1 Department of Psychiatry, Social Psychiatry and Psychotherapy, Hannover Medical School, Hannover, Germany; 2 Département de Neurologie, Assistance Publique-Hôpitaux de Paris (AP-HP), Hôpital de La Pitié-Salpêtrière, Paris, France; 3 Department of Pediatrics, Copenhagen University Hospital - Herlev and Gentofte, Herlev, Denmark; 4 Department of Clinical Medicine, University of Copenhagen, Copenhagen, Denmark; 5 Department of Health Sociology, Education and Medical Communication, Institute of Mother and Child, Warsaw, Poland; 6 Evelina London Children’s Hospital, GSTT and Kings College London, London, United Kingdom

**Keywords:** coprolalia, coprophenomena, differential diagnosis, functional neurologic disorder, functional tic-like behaviors, Non-Obscene Socially Inappropriate Symptoms, Tourette syndrome

## Abstract

Coprolalia is a relatively rare type of complex vocal tic in people with Tourette syndrome (TS). In the general population, however, it is believed that coprolalia is the most typical and common symptom resulting in the stereotype of TS as the “swearing disease”. Swearing, however, also occurs as a “normal” expression of negative emotions and thus occurs more often in people with impulse control problems. It is believed that there is a continuum reaching from voluntary to involuntary swearing. Besides coprolalia, in TS, coprophenomena may also occur in the form of motor tics, called copropraxia. Furthermore, other socially inappropriate behaviors have been described such as making socially inappropriate comments and actions, called Non Obscene Socially Inappropriate symptoms or NOSIS. Only recently, coprolalia has been identified as a core symptom in functional tic-like behaviors (FTLB), a relatively new and escalating presentation of a functional movement disorder (FMD). Today it is well-established that FTLB and TS represent two distinct clinical entities, although FTLB is a common comorbidity in TS. The overlap of diseases and disorders with the more incongruent presentations of FMD are well-established clinical concepts in other neurological disorders. This raises the clinically important question as to whether coprolalia as a complex tic can be differentiated at all from “coprolalia-like” words as FTLB. Here, we want to introduce a novel concept regarding the classification of coprolalia, copropraxia, and NOSIS. We propose that it is important to consider the presence of obscene and/or socially inappropriate words, behaviors and/or insults as being more supportive of FTLB or a combination of both TS plus comorbid FTLB as opposed to being considered to be complex tic phenomena, since coprolalia and copropraxia are rare in TS. Given what we know about FTLB today, the symptom construct of NOSIS is probably not a separate symptom entity in TS. We therefore suggest that NOSIS should be preferable considered as comorbid FTLB. We believe that this novel re-conceptualization will lead us toward some explanations around inconsistent findings related to coprophenomena in TS. Furthermore, it is of high clinical relevance, since it opens wider management and treatment strategies including psychological interventions for people suffering from coprolalia, copropraxia, and NOSIS and will hopefully prevent these patients from inappropriate or even harmful treatments including invasive surgical therapies when being mistakenly classified as severely affected and treatment resistant patients with TS.

**Content Warning: This article includes language that some readers may find offensive.**

## Historical aspects of coprolalia in Tourette syndrome

In his original description from 1885 (1), Georges Gilles de la Tourette emphasized the triad of multiple tics, coprolalia, and echolalia as pathognomonic of Tourette syndrome (TS), although “only” five of his original nine cases (56%) exhibited coprolalia, the repetitive, involuntary unintentional use of obscene or socially inappropriate words. According to Singer (2), the first patient with TS and coprolalia reported in the medical literature was published by Itard in 1825 (3). He described the case of Madame D., later known as the Marquise de Dampierre, born on 22 August 1800. Among other symptoms, she presented with vocalizations such as barking, repeating words, uttering vulgar curses and obscene epithets as well as blunting expressions of judgment and unfavorable opinions on others (2, 4). Retrospectively, some of her behaviors have been interpreted as non-obscene socially inappropriate symptoms (NOSIS) instead of coprolalia (4).

According to a population-based study from Germany in 2024 (5), coprolalia appears to be widely perceived by the general public as one of the most typical and common manifestations of TS, despite occurring in only a minority of affected individuals. This false belief is supported by misrepresentations of TS in the media with an overrepresentation of coprolalia as a tic manifestation (6) contributing to misconceptions and stigmatization and resulting in the stereotype of TS as the “swearing disease” (5). This has also led TS to being a chronic condition with especially high levels of judgment targeting (6). As a consequence, both healthcare professionals and lay people sometimes erroneously believe that the presence of coprolalia is necessary to diagnose TS (6, 7).

## Swearing

Since the early reports by Itard (3) and Gilles de la Tourette (1), coprolalia has been a perceived prominent symptom of TS. Although primarily associated with TS, the involuntary shouting of obscene words and swearing has also been described in other neurological disorders such as fronto-temporal dementia, epilepsy, structural brain lesions as well as neurodegenerative and autoimmune disorders (8, 9).

However, swearing also occurs in “normal” human communication in healthy people: on average around 0.5% of the daily spoken content includes taboo words (8). “Normal” swearing serves intra- and inter-personal functions including the expression of negative emotions such as anger, frustration, aggression, and pain (8) and is positively correlated with extraversion and hostility (10). Accordingly, verbal outbursts of taboo and swear words occur more often in people with impulse control problems, for example in conduct disorder (CD) or attention deficit/hyperactivity disorder (ADHD) (11). In general, it is believed that there is a continuum reaching from involuntary to unvoluntary to voluntary swearing (8).

At the same time, also beneficial aspects of swearing have been described resulting in enhanced performance by promoting psychological states conducive to maximizing effort and overcoming internal constraints (12). In addition, swearing has been shown to increase pain tolerance, to reduce perceived pain (13), and to have stress-reducing or cathartic effects (14). However, there are no descriptions available on such kind of subjective relief of coprophenomena in people with TS besides the well know transient relief following the execution of a tic (15).

Finally, it is important to keep in mind that the definition of a word as a swear word is not defined by its lexical content alone but strongly depends on the subjective definition and interpretation as well as on socio-cultural aspects. Thus, any listing of such words should be interpreted within its historical and sociolinguistic context, since the perceived offensiveness and taboo status of particular expressions is dynamic and varies according to cultural, linguistic, generational, interpersonal, and situational context. Due to an increasing normalization of profanity in everyday communication, a word historically classified as obscene, may today no longer be perceived as markedly inappropriate in many settings. Thus, a 2013 analysis of language use on Facebook should therefore be evaluated in light of this context, when classifying “shit”, “fuck”, “damn”, “bitch”, and “crap” as the five most frequently used English swear words at that time (16).

## Tourette syndrome

TS is a heterogeneous neurodevelopmental disorder in which clinical phenotype extends considerably beyond the presence of motor and vocal tics. Tic severity is inherently variable across time and context, while premonitory urges have been associated with tic severity, comorbidities, and quality of life (17, 18). Tics typically begin in childhood, with simple motor tics commonly preceding simple vocal tics; over time, the tic repertoire may change and become more complex, although the sequence and clinical course vary substantially between individuals (19). Vocal tics may range from simple sounds, such as sniffing, throat clearing, coughing, or grunting, to more complex vocalizations involving words or phrases (19). Coprolalia therefore represents one possible complex vocal manifestation within a much broader and dynamically evolving tic repertoire rather than a defining or obligatory feature of TS. The clinical burden of TS is further shaped by frequently associated neuropsychiatric and behavioral manifestations. ADHD and obsessive-compulsive symptoms/disorder (OCS/OCD) are particularly common, while anxiety, depression, learning difficulties, social communication difficulties, and episodes of disproportionate anger or rage may also occur (20).

## Coprolalia in Tourette syndrome

In the context of TS, coprophenomena are conceptualized as specific types of complex vocal and motor tics and include the exhibition of non-intentional obscene and socially inappropriate verbal expressions (coprolalia) or gestures (copropraxia). However, despite being generally classified as complex tics, there are no clear or unique definitions of coprophenomena. In general, coprolalia is characterized according to the interaction between content, context, phenomenology, and the individual’s broader clinical presentation rather than by reference to a predetermined list of “prohibited words”. According to Freeman et al. (21), coprophenomena have a “vulgar (obscene), religious (profane), or racially or ethnically insulting content, not expressed out of immediate anger or frustration, or solely for purposes of emphasis”.

In their pioneering 1988 monograph on TS, Shapiro et al. (22) used the term “coprophilia” as an umbrella term encompassing a spectrum of socially unacceptable verbalizations and actions, including coprolalia and copropraxia. Worth mentioning, this historical usage should not be confused with the contemporary meaning of coprophilia referring to a sexual/paraphilic interest in feces. The terminology used by Shapiro et al. was subsequently discussed by Kurlan et al. (23), who described the historical construct of “coprophilia” as encompassing not only obscene expressions and gestures but also racial slurs, religious improprieties, political innuendos, derogatory remarks, use of another person’s name, and other socially unacceptable or forbidden expressions. In a sample of 213 mostly adult patients with TS, Shapiro et al. documented 101 different coprolalic words and phrases. They described four-letter words, such as “fuck” (25%) and “shit” (17%), as the by far most common words, although also long phrases such as “fuck you, you mother-fucking, cocksucker, cunt” were documented.

In another pioneering book published in 1990, Comings defined coprolalia as “compulsive swearing” that - in contrast to “normal” swearing - occurs in a “compulsive, repetitive, almost ritualistic fashion” (24). Based on his experience with more than 1,400 cases, he also described the word “fuck” as the most common coprolalic word followed by “shit” and “piss”. In 1984, Goldenberg et al. (25) listed the following words as most common coprolalia in patients with TS in the US: “fuck”, “shit”, “bitch”, “asshole”, and “bastard”.

In the same year, Lee et al. (26) published one of the first larger case series from the UK including 53 patients and found the following words with sexual connotations most frequently (in descending frequency): “fuck”, “cunt”, “bastard”, “piss”, “sod”, “cock”, and “shit”. In addition, the authors described that swear words were typically uttered in a loud sharp tone, which contrasted with the otherwise normal prosody. When occurring during a conversation, coprolalia typically occurred during pauses between sentences. However, patients often tried to down-grade the impact of the words by using euphemisms or thinly disguised neologisms. Swear words were mainly uttered at home or in public lavatories (27).

In line with Lee et al.’s description (27), Müller Vahl (28) also characterized coprolalia more like an interjection with a loud, inarticulate, and altered tone of voice than as a part of a sentence. Patients often seem to try to disguise, deflect, shorten, or interrupt coprolalic words.

## Copropraxia in Tourette syndrome

In contrast to coprolalia, surprisingly, there are much less detailed descriptions on copropraxia in the literature. According to the case series published in 1984 by Lee et al. (27), most common obscene gestures were the palm-backed V sign, the forearm jerk with the fist clenched, the clenched fist with an extended index finger, and simulation of masturbation with rhythmical hand movements and pelvic thrusting. In addition, they reported that non-obscene gestures were common such as the thumbs up and victory V sign. In 1990, Comings (24) only briefly described copropraxia as “dirty movements” primarily in form of the “excessive use of the finger sign”. In 1988, Shapiro et al. (22) found copropraxia in 13% of their sample, most commonly with “touching or pointing to one’s own genital area or the genital area of another person”, “obscene finger gesture”, and “masturbatory movements”.

## Non Obscene Socially Inappropriate symptoms (NOSIS) in Tourette syndrome

In 1996, Kurlan was the first to describe NOSIS - in addition and in contrast to coprolalia and copropraxia - in patients with TS defined as socially inappropriate behaviors such as insulting others, making other socially inappropriate comments and socially inappropriate actions (23). In 2013, Eddy and Cavanna (29) performed a large prospective study on NOSIS in TS and found at least one of the following types or corresponding urges in 40/60 patients: (i) actions such as throwing a chair in church, kissing the teacher, and breaking children’s toys (50%), (ii) insults such as insults about height, weight, race, physical appearance, intelligence, and body odor (44%), and (iii) other remarks such as urges to shout ‘bomb’, ‘swine flu’, ‘Taliban’, or ‘I’ve got a gun’, urges to shout criticism about the food in a restaurant, or to make offensive remarks during a radio broadcast or public speech (38%). Although coprolalia and NOSIS overlap, some researchers have outlined the importance of differentiating them, suggesting NOSIS being classified as behaviors which convey contextually relevant information in an inappropriate way, e.g. shouting “bomb!” in a public place or making insulting remarks about a person’s physical appearance (8).

## Prevalence of coprophenomena in Tourette syndrome

In the literature, the frequencies of reported coprophenomena are extremely variable, ranging from 4–74% for coprolalia and 2–25% for copropraxia (21, 30). According to a review by Robertson et al. (31), coprolalia occurs in about 10–15% of all patients with TS and in about 30% of clinic populations. There has been much speculation about how to best explain wide ranges reported in the literature. Firstly, it has been suggested that different samples have been included, since sex, age, and overall disease severity might have an impact on prevalence rates (21). Tics have a clear age dependency and typically increase in severity during late childhood or early adolescence, and subsequently decrease substantially in many individuals during adolescence and early adulthood (32), but in the majority of patients persist into adulthood (26, 33, 34). Consequently, age may have an influence on frequency and phenomenology of coprophenomena (35). Secondly, larger samples are often studied in highly specialized TS centers, introducing bias towards more severely impacted groups. Thirdly, it can be assumed that for many years, patients with only mild tics and so called “TS only” (without comorbidities) (36) were heavily underdiagnosed resulting in an overestimation of coprophenomena, since a correlation has been described between the occurrence of coprophenomena and tic severity and number of comorbid conditions (21). Fourthly, it has been speculated that cultural influences have an impact on prevalence rates of coprophenomena (2, 25). On one hand, it has been suggested that, similar to “normal” swear words (9), it is culturally determined whether a tic and words or gestures, respectively, qualify as coprophenomena (37). On the other hand, it has been discussed whether prevalence rates indeed differ depending on the culture and/or the respective area in the world (38). Today, however, most researchers believe that there are *no* substantial racial, ethnic, or cultural differences in the clinical characteristics of TS (27, 39, 40) with studies concluding that there is a “remarkable degree of cross-cultural uniformity” (27). However, there are reports with clear variations of coprophenomena across cultures (38, 41). For example, in Japan, low rates of coprolalia of only 4 to 11% have been described, while in New Zealand and Hong Kong, respectively, reported rates are 46% to 60%, although we note data was published within a relatively narrow time period (between 1979 and 1994) (30). In 2008, Eapen and Robertson published a study comparing clinical presentation of people with TS in two distinct populations, the UK and the United Arab Emirates (41). While sample sizes were relatively small (N = 35, each), patients had been recruited in a similar time period (UK: 2004-2005, United Arab Emirates: 2001-2005). Interestingly, rates of both were significantly greater in the UK with coprolalia (UK: 25.7%, United Arab Emirates: 8.6%) and copropraxia (UK: 11.4%, United Arab Emirates: 2.9%).

Fifthly, it can be speculated that changes in definition of TS resulted in the inclusion of different samples. For example, in DSM-III (valid from 1980-1987), criteria for TS were stricter compared to DSM-5, with (i) age at onset between two and 15 years (instead of 18 years), (ii) presence of *multiple* (instead of one) vocal tics, and (iii) requirement of tic suppression for minutes to hours. Similarly, in DSM-IV (valid from 1994-2000), an impairment criterion was included with tics needing to “cause marked distress of significant impairment in social, occupational or other important areas of functioning” (42). Contrary to expectations, however, there is no clear relationship between reported prevalence of coprolalia and the year of investigation and consequently also not the DSM version valid at the respected time. For example, in the 1980s and 1990s, several investigators reported high prevalence rates of coprolalia ranging from 28-50% (for review see (21)), while others found much lower numbers, for instance, in 1982, Nomura et al. (43) reported a rate of 4% out of 100 patients in Japan. Similarly, only recently, in 2023 and 2025, respectively, Nilles et al. reported rather low prevalence rates of 5% for coprolalia and 3% for copropraxia in children or adolescents with tic disorders (19) and of 8% for coprolalia and 3% for copropraxia in adults (44) in patient groups from Canada and France. **Table 1** shows a summary of prevalence rates of coprolalia and copropraxia in larger patient groups depending on valid DSM versions between 1982 and 2025.

**Table 1:** Prevalence rates of coprolalia and copropraxia depending on year and valid DSM version.

Year	DSM version	Author	Country	Sample size (N)	Coprolalia	Copropraxia
1982	III	Nomura et al. (45)	Japan	100	4%	–
1985	III	Comings & Comings (46)	USA	250	3%	–
1994	IV	Goldenberg et al. (34)	USA	112	8%	–
2009	IV-TR	Freeman et al. (7)	International	597	18.5% 8 (children: 16.9%, adults: 27.5%)	5.7% (children: 5.8%, adults: 5.5%)
				3 largest sites: coprophenomena (not differentiated between coprolalia/-praxia): 31.9% (n=94), 14.8% (n=134), 9.3% (n=215)		
2013	5	Eddy & Cavanna (47)	UK	60	33%	–
2014	5	Kobierska et al. (48)	Poland	168	25%	–
2015	5	Eapen & Robertson (49)	UK	400	39%	–
2016	5	Sambrani et al. (27)	Germany	1032	24%	15.5%
2023	5-TR	Nilles et al. (23)	Canada/France	–	5% (children only)	3% (children only)
2025	5-TR	Nilles et al. (29)	Canada/France	–	8% (adults only)	3% (adults only)

Taking together, prevalence rates for coprolalia encompass an extremely wide range, which in our opinion cannot be reasonably explained by the year of investigation or changes in DSM criteria or clinical characteristics or cultural influences.

## Coprolalia in functional tic-like behaviors

In 2016, for the first time, coprolalia has been proposed as a possible symptom of functional neurological disorder (FND) (50). Shortly thereafter, and peaking during the COVID-19 pandemic, researchers from all over the world described samples of children, adolescents, and adults with functional tic-like behaviors (FTLB) as a relatively new and escalating presentation of a functional movement disorder (FMD), who typically presented with coprolalia and other complex vocalizations such as insults and comments (51, 52). FTLB are characterized by mainly sudden-onset movements and/or vocalizations often after the age of 12 years, most typically presenting with complex and stereotypical movements involving the arms and hands (not following the typical rostro-caudal distribution of primary tics), self-hitting, “tic attacks”, and enunciations in form of obscene utterances and insults, as well as nonsensical, non-obscene syllables, words, long phrases (> three words), sometime directed to a specific person, or out-of-context words. Behaviors typically have a clear context dependency (51, 53, 54). Examples for coprolalic statements in patients with FTLB include exclamations such as “fuck you”, “asshole”, “fuck off, you bitch”, “fuck off right now”, “you chunky ass bitch”, “Heil Hitler”, “gay sow”, “shit turks”, and “faggot” or even more complex vocalizations such as “sex with children”, “I have not offended you, you fuck cunt”, “you are so ugly”, and “I am a pedophiliac” (50, 55, 56).

Based on a large number of clinical studies on FTLB published since 2020, a group of experts defined diagnostic criteria for FTLB (53), that have proven to be specific in distinguishing between primary tic disorders/TS and FTLB (57). At the same time, Trau et al. (58) independently suggested very similar diagnostic criteria for FTLB. Among other characteristics, they proposed at least two of the following seven phenomena for diagnosing FTLB: (i) tic attacks, (ii) blocking tics, (iii) frequent coprolalia or other coprophenomena, (iv) broad spectrum of word use or frequent coprolalia, (v) self-injurious tics or tics injurious to others, (vi) throwing, or (vii) presence of other functional neurological symptoms.

## Differentiation between Tourette syndrome and functional tic-like behavior

Despite these useful diagnostic criteria, differentiating primary tics from FTLB can still be challenging. Remarkably, even among expert clinicians the level of diagnostic agreement is low when using only videos, but can be increased by providing additional clinical information such as symptom semiology, age at onset, presence of premonitory urges, suppressibility, triggers, and changes over time (59). Unfortunately, none of the multiple characteristics of primary tics including waxing and waning, suppressibility, and premonitory urge, taken on its own, allows for a reliable distinction from FTLB. However, all these characteristics should also be applied, when trying to differentiate between coprolalia and copropraxia as complex tics and coprophenomena in the context of FTLB (46, 47). Accordingly, it is not surprising that currently a substantial number of people with FTLB are misdiagnosed as “late-onset treatment refractory TS” (54). In a relevant publication from 2023, Nilles et al. (19) asked the simple question “Have we forgotten what tics are?”. Based on a large sample (N = 203 children and adolescents with primary tic disorders), the authors reminded us that tics are typically “simple movements” such as eye blinking, head jerks, and nose and mouth movements as well as “simple vocalizations” such as throat clearing and sniffling. According to DSM-5, tics – in contrast to FTLB - are defined as sudden, rapid, recurrent, non-rhythmic movements or vocalizations. Motor tics – but not FTLB - typically follow a rostro-caudal distribution.

Only recently, it became clear that FTLB is a common comorbidity in TS (48, 49) making the differentiation between both even more difficult. Unsurprisingly, coprolalia was found to be significantly more likely in patients with TS plus FTLB compared to those with TS without comorbid FTLB (48).

## Novel concept of coprolalia in Tourette syndrome

Since shouting obscene words, insults, and other socially inappropriate words have been identified as typical symptoms of FTLB and coprolalia is a rare, but well-known symptom in TS, the important question arises as to whether coprolalia as a complex tic can be differentiated at all from “coprolalia-like” words as FTLB. This is even more relevant in patients suffering from both TS and comorbid FTLB. Some researchers suggested time at onset (60), or the absolute number and kind of swear words as differentiating factors (49). Furthermore, patients’ descriptions of premonitory urges have been suggested as being useful to help differentiate between TS related tics and FTLB (49, 50), while others found no relevant differences (61).

Here, we want to introduce a novel concept regarding the classification of coprolalia, copropraxia, and NOSIS within the TS spectrum. We propose that it is important to consider the presence of obscene and/or socially inappropriate words, behaviors and/or insults as being more supportive of comorbid FTLB as opposed to being considered to be complex tic phenomena. This proposal is based on the following: Firstly, it is well-known that people with TS exhibit, by far, more motor compared to vocal tics. However, with coprophenomena, the reverse is true, with vocal expressions being much more common than copropraxia with a consistently reported prevalence of coprolalia being 3–4 times higher than that of copropraxia (21, 37).

Secondly, besides the fact that people with TS have by far more motor than vocal tics, in addition, chronic tic disorder other than TS occur much more frequently in form of chronic *motor* than chronic *vocal* tic disorder (28). In other words, motor tics alone (without vocal tics) occur much more frequently than the reverse case. However, with coprophenomena the opposite is true: coprolalia has been found to occur much more likely as the exclusive expression of coprophenomena than copropraxia. In large cohorts of patients, there was not a single adult who exhibited copropraxia without coprolalia (21, 62).

Thirdly, as outlined above, prevalence rates of coprophenomena reported in the literature largely vary without any clear explanation, while for other TS-related aspects such as age at onset and sex ratio there is a remarkable degree of cross-cultural uniformity. Against this background it is plausible to speculate that differences in prevalence rates of coprophenomena are due to comorbid FTLB rather than TS, since FTLB is strongly influenced by context, culture, and even language (51).

Fourthly, several studies investigated clinical correlations between coprolalia and other clinical symptoms in samples of people with TS. For example, Baizabal-Carvallo and Jankovic (63) described not only a significant association between “complex phonic tics” and the presence of trunk tics, but also between coprophenomena and impulsive behavior, rage outbursts, and intrusive behavior such as forced touching. Taking into account what we currently know about FTLB, it can be speculated that those symptoms associated with coprophenomena also represent comorbid FTLB rather than TS. Similarly, Freeman et al. (21) reported a “robust” association of coprophenomena with the number of non-tic repetitive behaviors including self-hitting as well as spitting and inappropriate sexual behavior, which all have been described as common behaviors in the context of FTLB (49, 51, 56, 57, 64). In line with this data, Kano et al. (65) found more frequently self-injurious behaviors and compulsions in patients with TS who also suffered from coprolalia. Similar to data described by Freeman et al. (21), Eapen and Robertson (36) found that coprolalia was present in 39% of patients with “TS plus” (with comorbidities) compared to 0% in “pure TS” (“TS only”, without comorbidities). At the same time, they reported an association between coprolalia and tic severity according to the Yale Global Tic Severity Score (YGTSS) (66). While so far associations between coprophenomena and both tic severity and the number of comorbidities have been explained by the fact that coprophenomena occur with increasing severity of the disease, in contrast, we argue that people with FTLB show not only higher scores on the YGTSS than people with primary tic disorders, but also have more frequent comorbidities (64).

Fifthly, the established definition of NOSIS as “socially inappropriate behaviors such as insulting others, making other socially inappropriate comments, and socially inappropriate actions” (23) including clear context-dependent statements (29) is very similar to today’s definition of FTLB. This was proposed at a time, when it was believed that FTLB was a very rare presentation of FND and combinations of TS and FTLB hardly existed. In other words, it is challenging to differentiate “so called” NOSIS from complex nonsensical, coprolalic, and socially inappropriate utterances in the context of FTLB. Given what we know about FTLB today, the symptom construct of NOSIS is probably not a separate symptom entity in TS. We therefore suggest that, in many cases, NOSIS could be preferable considered as comorbid FTLB.

Sixthly, with respect to coprolalic words, already in the past, a clear context-dependency, cultural influences, as well as long phrases with more than three words have been described in people diagnosed with TS. As a typical example, the provocative slogan in the post-cultural revolution era of China during the 1980s “down with Chou En-lai” (note: Chou En-lai was communist China’s first and longest-serving leader) of a young girl is reported (38, 67). It can be assumed that from today’s perspective, the majority of TS experts would classify such exclamations as FTLB rather than a tic.

## Comorbid functional tic-like behavior in Tourette syndrome

Although Georges Gilles de la Tourette speculated about an “organic origin” of tics (68), about 80 years later, this assumption was supported for the first time by robust data, when it was demonstrated that treatment with haloperidol resulted in a marked reduction of tics (22, 69). In the decades before 1960, there had been repeated speculation as to whether tics are “psychogenic” in nature. However, we now know that TS is a neuropsychiatric/neurodevelopmental disorder - with clear underlying mechanisms – but often occurs together with a functional presentation in form of FTLB (48, 49).

The overlap of diseases and disorders with the more incongruent presentations of FND are well-established clinical concepts in other neurological disorders such as epilepsy with a mean frequency of additional “psychogenic non-epileptic seizures” (PNES) – today called functional/dissociative seizures (FDS) - of about 12%, as well as movement disorders such as Parkinson’s disease with a prevalence of FMD comorbidity of more than 7% (70). Although the prevalence rate of comorbid FTLB in TS is currently unknown, from recent studies it is suggested that FTLB is a common comorbidity in TS and occurs much more frequent than previously thought (48, 49).

Current neurobiological models suggest potentially different mechanisms underlying primary tics and FTLB, although no pathophysiological mechanism specific to coprophenomena has yet been established. In TS, converging neuroimaging, neurophysiological, pharmacological, and experimental evidence implicates dysfunction within cortico-basal ganglia-thalamo-cortical circuits and their connected regions involved in motor selection, action control, and inhibition (71, 72). Alterations in several neurotransmitter systems, particularly dopaminergic, GABAergic, and glutamatergic signaling, have been implicated in tic generation (73). Primary complex tics including coprolalia and copropraxia would therefore be expected to arise within these neurodevelopmentally altered tic-generating networks. Interactions between motor circuitry and limbic, language, and inhibitory-control networks involved in emotionally salient verbal and behavioral output have been proposed (8, 36), but a specific neural signature of coprophenomena has not been demonstrated.

FTLB, in contrast, are conceptualized within contemporary neurobiological models of FND. These models emphasize altered interactions among attention, expectations and predictive processing, sense of agency, interoception, emotional and salience processing, and motor control (74). Neuroimaging studies of FMD suggest alterations across distributed networks involving motor and premotor regions, temporoparietal areas, insula, amygdala, and other regions involved in salience, emotion and self-agency rather than dysfunction of a single motor pathway (74). In FTLB specifically, contextual factors, symptom-directed attention, expectations, and social modeling may additionally contribute to symptom selection and expression (75), potentially contributing to the prominence of complex and context-dependent vocalizations and socially salient behaviors. However, these mechanisms have not been demonstrated specifically for coprophenomena-like symptoms in FTLB.

Thus, although the current neurobiological frameworks underlying primary tics and FTLB differ conceptually, available evidence does not permit differentiation between TS-related and functional coprophenomena on neurobiological grounds in an individual patient.

## Conclusion

In summary, the presence of coprophenomena should be seen as a strong indication to consider the presence of FTLB or a combination of both TS plus comorbid FTLB. We would argue that coprophenomena no longer should be considered just typical phenomenon of TS. According to recent studies - and in contrast to common beliefs - coprolalia and copropraxia are rare in TS.

Based on current definitions and data, in the authors’ opinion, it is not always possible to differentiate NOSIS from complex nonsensical, coprolalic, and socially inappropriate utterances in the context of FTLB. Even “normal” swearing as an expression of negative emotions, which also more frequently occurs in patients with TS due to comorbid conditions associated with impulse control problems such as CD and ADHD can be challenging to distinguish but could also be seen as NOSIS. Following Occam’s razor - also referred to as the “principle of parsimony” - we therefore suggest considering reframing the concept of NOSIS in some with TS, as FTLB in accordance with the rule to prefer the simpler explanation over more complex ones with the need of clear scientific proof for the “more complicated assumption” as opposed to the simpler one.

We believe that this novel re-conceptualization will lead us toward some explanations around the varying prevalence rates, cultural differences, and phenomenology of coprophenomena in the context of TS. Furthermore, it is of high clinical relevance, since it offers new perspectives in evaluation, understanding and importantly opens wider management and treatment strategies for people suffering from coprolalia, copropraxia, and NOSIS. We are yet to fully understand the underlying neurobiological basis of FND in general and FTLB in particular. It would be especially interesting and important for future work to examine if the neurobiological mechanisms of coprophenomena in TS and FTLB overlap.

From a clinical perspective, the proposed reconceptualization may reduce diagnostic anchoring on severe or treatment-refractory TS when prominent coprophenomena actually reflect comorbid FTLB. Recognizing a functional component may prevent unnecessary escalation of tic-directed pharmacotherapy or inappropriate invasive interventions and may instead facilitate psychoeducation, behavioral and psychological treatment, modification of reinforcing factors, and multidisciplinary FND-oriented care. In patients with both TS and FTLB, symptom-level differentiation may enable a more individualized treatment strategy directed separately at neurodevelopmental tics and FTLB. Correct classification of coprophenomena may therefore improve treatment selection and, when symptoms are functional, provide an opportunity to target potentially modifiable mechanisms associated with FTLB.

Taken together, these considerations suggest that coprophenomena should not be defined or diagnostically attributed on the basis of obscene content alone. We propose a multilevel clinical assessment that distinguishes (i) content - what is said or performed and how, and whether it is considered socially inappropriate within the relevant cultural and interpersonal context; (ii) phenomenology - whether the behavior has typical characteristics of primary tics; (iii) longitudinal context - whether age and mode of onset and the course of symptoms is typical for primary tics and, if applicable, in relationship to other motor and vocal tics; and (iv) diagnostic attribution - whether the overall presentation is best explained by TS or another primary tic disorder, FTLB, their coexistence, or another behavioral phenomenon. This distinction is particularly important because neither the presence of a swear word nor recognition of an utterance as pathological establishes its underlying mechanism. Accordingly, prominent coprophenomena should be regarded as a signal for careful phenotypic and diagnostic characterization rather than as a diagnostic proxy for TS or FTLB.

## Data Availability

The original contributions presented in the study are included in the article/supplementary material. Further inquiries can be directed to the corresponding author.
